# Extirpation of Larynx and Part of Tongue

**Published:** 1876-04

**Authors:** 


Extirpation of the Larynx with the Hyoid Bone and Part of the Tongue, Pharynx, and Œsophagus. (V. Langenbeck : *Centralblatt fur die Med. Wissenschaften*, No. 51, 1876). —In November, 1874, V. L. performed tracheotomy upon the patient, a tailor, aged 57 years, on account of dyspnœa of high grade, caused by carcinoma of the larynx. The patient, who at that time was unwilling to submit to any further operation, returned in July, 1875, and then it was resolved to perform extirpation of the larynx and of such portions of the neighboring parts as had been invaded by the new growth. After the patient had been brought under the influence of chloroform, the fistula of the trachea was enlarged downwards, and, a canula having been introduced, a T-shaped incision was made through the skin, the perpendicular limb of which was in the median line of the neck, and extended almost to the trachael opening. The skin was then dissected away, and the submaxillary, lymphatic, and salivary glands which were involved were removed. The muscles of the floor of the mouth were divided, and the arteries of the tongue ligated. The pharynx was then opened, the posterior part of the tongue cut through, and the anterior wall of the pharynx and œsophagus removed.

The external carotid artery was then ligated, and finally the trachea divided above the point at which the canula had been introduced. Forty-one ligatures were applied, and numerous muscles and nerves divided. No sutures were introduced, and the wound was dressed simply, but so as to prevent the entrance of fluids into the windpipe. At the time that the report of the case was made, eight days after the operation, no unfavorable symptoms had been noticed. The growth was found to be a carcinoma, which had started from the upper part of the larynx.

